# Whole-genome sequences of *Parageobacillus caldoxylosilyticus* strains isolated from soil in Japan

**DOI:** 10.1128/mra.00408-26

**Published:** 2026-05-29

**Authors:** Haruki Omichi, Kentaro Miyazaki, Hiroya Tomita, Kohsuke Honda

**Affiliations:** 1 Graduate School of Engineering, The University of Osaka 13013, Suita, Osaka, Japan; 2 International Center for Biotechnology, The University of Osaka 199496, Suita, Osaka, Japan; 3 Industrial Biotechnology Initiative Division, Institute for Open and Transdisciplinary Research Initiatives (OTRI), The University of Osaka 13013, Suita, Osaka, Japan; Montana State University, Bozeman, Montana, USA

**Keywords:** *Parageobacillus*, *Parageobacillus caldoxylosilyticus*, *Saccharococcus*, thermophile, complete genome sequence

## Abstract

Here, we present the complete genome sequences of four *Parageobacillus caldoxylosilyticus* strains isolated from soil in Japan. The strains showed ≥97.5% average nucleotide identity (ANI) with the *P. caldoxylosilyticus* type strain S1812^T^ and ≥97.0% ANI with each other.

## ANNOUNCEMENT

*Parageobacillus* (NCBI Taxonomy ID: 1906945) (1) is a genus of gram-positive, aerobic, spore-forming, non-motile, heterotrophic bacteria belonging to the Anoxybacillaceae (NCBI Taxonomy ID: 3120669) (2), which exhibits moderately thermophilic properties (1, 3). There are currently six valid species registered in the *Parageobacillus* genus (https://lpsn.dsmz.de/genus/parageobacillus), with *Parageobacillus thermoglucosidasius* serving as the type species (3). Complete genome sequences of 15 strains of *Parageobacillus* (https://www.ncbi.nlm.nih.gov/datasets/genome/?taxon=1906945) have been reported, including 5 strains that we isolated from Japanese soil (4). Interestingly, antiSMASH analysis (5) has identified biosynthetic gene clusters within these genomes, shedding new light on the potential of secondary metabolism in this group of thermophiles (6). For example, nonribosomal peptides such as bacillibactin (7) and ribosomally synthesized and post-translationally modified peptides such as parageocin I (8) have been identified in this genus.

Samples were collected directly from the soil surface of a municipal park in Hannō City, Saitama Prefecture, using a toy shovel. The samples (1 g) were suspended in 10 mL of distilled water and spread over TT (8 g L^−1^ tryptone, 4 g L^−1^ yeast extract, and 2 g L^−1^ NaCl) medium plates solidified with agar (1.6 g L^−1^). After incubating the sample at 65°C for 15 h, several well-separated single colonies were isolated. We performed colony PCR to analyze the 16S rRNA gene using the Bac8f(C) and UN1542r primers (9). Whole-genome analysis was performed on four strains, designated NDK1, NDK2, NDK3, and NDK6, which were presumed to belong to the genus *Parageobacillus*. Here, we report the complete genome sequences of these strains.

To prepare genomic DNA, cells were grown in 5 mL of LB (10 g L^−1^ tryptone, 5 g L^−1^ yeast extract, and 10 g L^−1^ NaCl) broth at 65°C for 15 h with vigorous shaking (200 rpm). Genomic DNA was purified using a Blood and Cell Culture DNA Midi Kit (Qiagen). For long-read sequencing, unsheared genomic DNA (1 µg) was treated with a short-read eliminator kit (Circulomics) to remove <10 kbp fragments, and a library was constructed using a ligation sequencing kit (Oxford Nanopore Technologies [ONT]). Sequencing was performed with a GridION X5 system on a FLO-MIN106 R9.41 revD flow cell (ONT). Base calling was conducted using Guppy v.4.0.11. The raw sequencing data (Table 1) were filtered (*Q* < 10; length <1,000 bases) using NanoFilt v.2.7.1 (10). For short-read sequencing, a library was constructed using an MGIEasy FS PCR Free DNA Library Prep Set (MGI) with a ∼400- to 500-bp insert. Paired-end sequencing (2  ×  150 bases) was then performed on a DNBSEQ-400 instrument (MGI). The raw sequencing data (Table 1) were filtered (*Q* < 30; length < 20 bases) using fastp v.0.20.1 (11). The trimmed long- and short-read data were assembled using Unicycler v.0.4.8 (12), and the assembly was polished using Pilon v.1.24 (13); the circularity was confirmed using Unicycler v.0.4.8 (12). Each strain contained a single circular chromosome and multiple circular plasmids. Completeness was assessed using CheckM v1.2.2 (14), implemented in the DFAST v.1.6.0 (15). For all software, default parameters were used.

**TABLE 1:** Sequencing metrics for the four *Parageobacillus caldoxylosilyticus* strains in this study

		MiSeq (short read)			GridON (long read)				Complete genome				
	BioSample accession no.	No. of paired-end reads	Total length (Mb)	Accession no.	No. of reads	*N*_50_ (bases)	Total length (Mb)	Accession no.	Length (bp)	G + C (%)	No. of cds	Accession no.	Avg. read depth (×)
NDK1	SAMD01829901	12,257,368	1,838.6	DRR915103	152,299	92,834	579.5	DRR915107					
Chromosome									3,750,003	44.3	3,732	AP046161	808.8
pScNDK1b									52,590	40.3	54	AP046162	3,103.1
pScNDK1c									45,671	40.1	46	AP046163	2,872.8
pScNDK1d									44,019	40.3	43	AP046164	358.9
pScNDK1e									38,136	41.5	66	AP046165	1,675.9
pScNDK1f									15,734	40.1	23	AP046166	11,524.0
pScNDK1g									7,899	42.6	9	AP046167	7,822.7
NDK2	SAMD01829902	5,059,686	759	DRR915104	675,617	62,657	1,746	DRR915108					
Chromosome									3,780,367	44.3	3,761	AP046168	356.7
pScNDK2b									69,458	40.2	63	AP046169	1,047.2
pScNDK2c									51,736	38.7	60	AP046170	1,695.9
NDK3	SAMD01829903	5,669,787	850.4	DRR915105	898,682	82,972	1,701.3	DRR915109					
Chromosome									3,785,891	44.3	3,712	AP046171	435.9
pScNDK3b									50,949	39.3	53	AP046172	829.9
NDK6	SAMD01829904	4,878,791	731.8	DRR915106	400,233	239,302	3,118.4	DRR915110					
Chromosome									3,762,496	44.6	3,800	AP046173	323.1
pScNDK6b									81,276	40	79	AP046174	1,125.0
pScNDK6c									67,236	39.9	78	AP046175	474.4
pScNDK6d									35,627	37.4	45	AP046176	1,076.4
pScNDK6e									15,733	40.1	24	AP046177	5,128.4

Automatic annotation was conducted using DFAST v.1.6.0 (15); genomic features are summarized in Table 1. Genomic-based taxonomic validation on the TYGS (16) server revealed that all four strains belong to *P. caldoxylosilyticus* (basonym *Saccharococcus caldoxylosilyticus*) (1, 17). Further JSpecies analysis (18) revealed that all strains exhibited an average nucleotide identity (ANI) of ≥97.5% with the *P. caldoxylosilyticus* type strain S1812^T^ (17) (GCF_019272935.1). The ANI between individual strains was ≥97.0%.

## Data Availability

All four *Parageobacillus* strains reported in this paper are associated with BioProject PRJDB12551. BioSample accession numbers, genome sequences, and raw sequencing data are available under the accession numbers listed in Table 1.
